# Discrimination of Small Forms in a Deviant-Detection Paradigm by 10-month-old Infants

**DOI:** 10.3389/fpsyg.2019.01032

**Published:** 2019-05-14

**Authors:** Marcus Lindskog, Maria Rogell, Ben Kenward, Gustaf Gredebäck

**Affiliations:** ^1^Department of Psychology, Uppsala University, Uppsala, Sweden; ^2^Department of Psychology, Health and Professional Development, Oxford Brookes University, Oxford, United Kingdom

**Keywords:** geometry, eye-tracking, infants, deviant-detection, small forms

## Abstract

Using eye tracking, we investigated if 10-month-old infants could discriminate between members of a set of small forms based on geometric properties in a deviant-detection paradigm, as suggested by the idea of a core cognitive system for Euclidian geometry. We also investigated the precision of infants' ability to discriminate as well as how the discrimination process unfolds over time. Our results show that infants can discriminate between small forms based on geometrical properties, but only when the difference is sufficiently large. Furthermore, our results also show that it takes infants, on average, <3.5 s to detect a deviant form. Our findings extend previous research in three ways: by showing that infants can make similar discriminative judgments as children and adults with respect to geometric properties; by providing a first crude estimate on the limit of the discriminative abilities in infants, and finally; by providing a first demonstration of how the discrimination process unfolds over time.

## 1. Introduction

People process geometric information in a variety of situations in their day-to-day lives. It is used intuitively in such diverse situations as navigating a new city or identifying objects. Geometry is also taught already in the first years of school. The formal mathematical system of geometry that children first encounter is Euclidian geometry, which is an axiomatic system where theorems about two-dimensional (planar geometry) and three-dimensional (solid geometry) objects are derived from a small set of axioms. The intuitive use of Euclidian geometry is also found in cultures where it is not formally taught (Izard et al., 2011) and even in non-human animals (Spelke and Lee, 2012). The universal use of Euclidian geometry and the ease with which its general concepts are understood has lead philosophers and mathematicians to suggest that it comes naturally to the mind (see, e.g., Spelke et al., 2010). More recently, cognitive scientists have even suggested that Euclidian geometry as a mathematical system might have emerged as a result of evolutionary ancient core systems that capture fundamental Euclidian concepts (Dehaene et al., 2006). The idea of a dedicated system for Euclidian geometry is appealing, and several studies support it (Dehaene et al., 2006; Izard and Spelke, 2009; Spelke et al., 2010; Dillon et al., 2013; Bonny and Lourenco, 2015). There are still, however, several outstanding questions that could help us better understand the properties and processes of such a system. In the present study, we aim to bridge two previous lines of research conducted on preverbal infants and preschool children, respectively. More specifically, by using eye tracking, we present the first study assessing 10-month-old infants' ability to discriminate between small forms in a deviant-detection paradigm.

**Figure 1:**
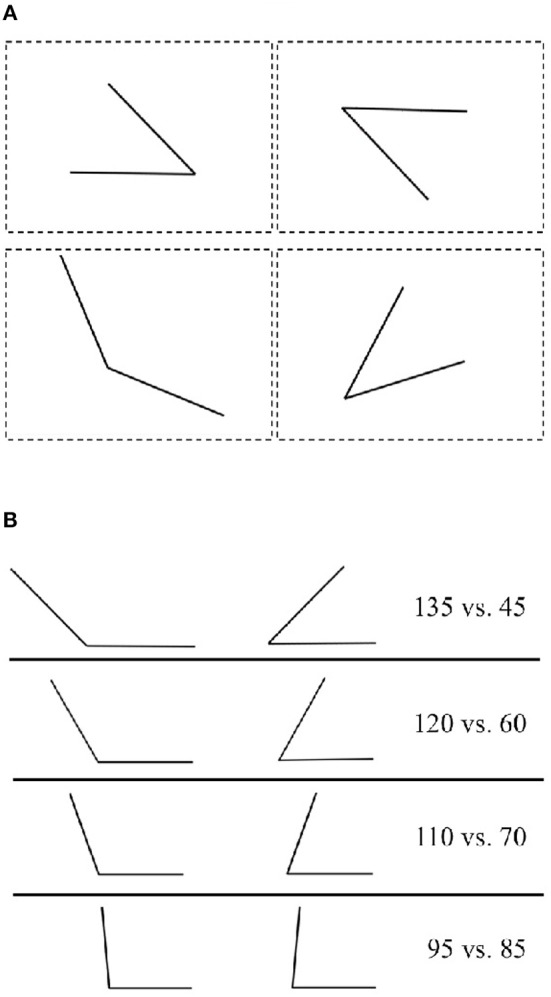
**(A)** Illustration of the organization of the array of four small forms presented on each trial with AOI:s superimposed as dashed rectangles. **(B)** Illustration of the small forms used in each of the four conditions.

Researchers have primarily relied on visual-form tasks to investigate a possible core system for geometry. In these tasks, participants are presented with small 2D visual forms (see Figure 1A for an example) and are expected to respond to or detect differences between them based on geometric properties. Early research combining visual-form tasks with habituation paradigms indicated that infants as young as a few days old form expectations about an invariant geometric property and dishabituate when these expectations are violated (Schwartz et al., 1979; Cohen and Younger, 1984; Younger and Gotlieb, 1988; Slater et al., 1991; Lourenco and Huttenlocher, 2008). Cohen and Younger (1984), for example, habituated 6- and 14-week-old infants to small forms consisting of two lines making a single angle. More specifically, infants were habituated to either a form with an angle of 45° or one with 135°. The orientation of the two lines was constant during habituation but differed for the two forms. During the test trials, infants were shown forms that corresponded to the habituation stimuli either with respect to angle size or orientation. The results showed that 14-week-old infants dishabituated to a change in angle size but not to a change in orientation. In contrast, 6-week-old infants showed the opposite effect; they dishabituated to a change in orientation but not to one in angle size. The authors concluded that there is a developmental shift in perceptual ability between 6 and 14 weeks of age.

Slater et al. (1991) repeated the experiments of Cohen and Younger (1984) with 3-day-old infants. Their results indicated, similar to the 6-week-old in Cohen and Younger (1984), that the infants dishabituated to orientation, but not to angle size when they were habituated with small forms of fixed orientation. Slater et al. (1991) also made one important extension to the studies by Cohen and Younger (1984). In addition to habituating infants to a form with a fixed orientation, they also carried out a condition where the angle size was fixed but where the orientation of the angle changed from trial-to-trial during habituation. When infants were habituated to these figures where the orientation also varied, they also dishabituated to angle size. Taken together, these findings indicate that infants can notice a change in an invariant geometric property that they have had the opportunity to extract over repeated presentations. That is, they can recognize which geometric property stays the same in a series of small forms. It should be noted that although both Cohen and Younger (1984) and Slater et al. (1991) manipulated the difference between the forms in terms of angle size, angle size was confounded with other metric aspects of the stimuli (e.g., endpoint distance or line length depending on what other metric aspect was controlled for). Accordingly, from these studies it is not possible to conclude which geometric property infants used to discriminate between the small forms.

While early studies on infants combined visual-form tasks with habituation paradigms, more recent research on older children and adults instead combines the idea of a visual-form task with a deviant-detection paradigm. In this paradigm (e.g., Dehaene et al., 2006), participants are presented with an array of small forms. All but one of the forms share a common geometric feature, for example, angle size. Participants' task is to identify which form deviates from the others. Research using this paradigm has shown that at least from the age of 4 years, children can reliably indicate which the deviant form is (Izard and Spelke, 2009; Dillon et al., 2013; Bonny and Lourenco, 2015). The deviant-detection paradigm can give additional insights into participants' ability to discriminate based on geometric cues over and above those provided by habituation paradigms. First, it makes it possible to manipulate both on which cue the deviant form is different and the degree to which it deviates, within subjects. Second, because the task is a decision task, it could also potentially be used to study the process that leads up to discrimination. The deviant-detection paradigm, however, has limitations when studying infants. More specifically, in its current form the paradigm requires participants to choose actively (e.g., by pointing), which form deviates. Consequently, the paradigm imposes a lower limit on participants' age.

### 1.1. The Present Study

Previous studies have thus indicated that infants as young as a few days old are sensitive to geometric properties and that children at least from the age of four can identify which object in a set of small forms has a deviant geometric property. Here, we aim to bridge these two findings to gain further insights into infants' ability to discriminate between small forms. More specifically, by using eye tracking, we assess 10-month-old infants' ability to discriminate between small forms based on geometric properties in a deviant-detection paradigm. In doing so, we extend previous research in two critical ways. First, although infants might be able to make such discriminations, it is possible that there is a limit to this ability concerning acuity. Here, we investigate this possibility by manipulating the difference between the presented small forms in terms of angle size. Second, by tracking gaze patterns throughout the stimulus presentation, we investigate how detection of a deviant visual form unfolds over time. To our knowledge, no previous study has examined the temporal structure or the limits of an ability to discriminate based on geometric properties.

## 2. Method

### 2.1. Participants

Twenty full-term 10-month-old infants (12 females, *M*_*age*_ = 309.1 days, *SD* = 6.6) participated in the study, and all participating infants were included in the final analysis. All infants were recruited from a midsized Swedish town. All parents gave written informed consent before the study, and the families were given a 100 SEK (approximately 10 euros) gift certificate in exchange for their participation. The regional ethics committee approved the study according to the 1964 Declaration of Helsinki.

### 2.2. Apparatus and Stimuli

Infants' gaze was recorded with a Tobii TX300 (Stockholm, Sweden) eye-tracker at 60 Hz while the stimuli were presented on a 19-in. screen with resolution 1,920 × 1,080. The stimuli consisted of two sets of eight images each, one set for each of the two conditions described below. Each image contained an array of four small forms arranged in a square pattern (see Figure 1A). The small-forms consisted of two connected lines, 6.2 cm in length (5.9 visual degrees), which formed an angle. Three of the forms were identical (base-line-forms) in terms of geometrical properties (angle, length, and/or enclosed area) while the fourth (target form) deviated. Each of the forms was placed in one of the quadrants, equidistant from the origin, in a Cartesian coordinate system. The orientation of each form in each image was set randomly. The entire image subtended 27 × 19 visual degrees and each angle subtended 12 × 8 visual degrees. Pilot data indicated that infants would not reliably attend to the stimuli for more than eight trials. To investigate a range of differences we, therefore, manipulated the difference between the target form and the base-line-forms, in terms of angle size, in a mixed design. Half of the participants saw *Large* angle differences that were manipulated within-subjects in two steps, 90° (135 vs. 45) and 60° (120 vs. 60). The other half saw *Small* angle differences, also manipulated within-subjects in two steps, 40° (110 vs. 70) and 10° (95 vs. 85). The location of the target angle (quadrant 1–4) and whether the target angle or the base-line-angle was larger was counterbalanced over trials.

Changing one geometric property, like angle size, inevitably leads to a change in other properties, such as the enclosed area and the distance between endpoints (see Table 1). The aim of the present study was not to determine which of these properties infants use to discriminate between small forms, but rather to investigate if they can discriminate at all between small forms that incorporate some of these aspects and how the discrimination process unfolds over time. Accordingly, to ease the presentation, we only present our manipulations in terms of angle size (see Figure 1B for an illustration).

**Table 1:** Angle size, enclosed area, and distance between endpoints for each of the small-forms displayed to the participants in each of the eight conditions.

**Condition**	**Angle (^**°**^)**	**Enclosed area (cm^**2**^)**	**Endpoint distance (cm)**
Small–10	85	19.15	8.38
Small–10	95	19.15	9.14
Small–40	110	18.06	10.16
Small–40	70	18.06	7.11
Large–60	120	16.65	10.73
Large–60	60	16.65	6.20
Large–90	135	13.59	11.46
Large–90	45	13.59	4.75

### 2.3. Procedure

Participants were seated approximately 60 cm in front of the eye-tracker in their caregiver's lap. The caregiver was instructed not to comment on anything presented on the screen. Following a standard 5-point calibration procedure (Gredebäck et al., 2009) infants were shown a 5-min long movie where the images described above were presented intermixed with various unrelated stimuli, not described here, that were part of other studies, and attention-grabbing movies. Participants were shown each of the four small-form images in a condition once, for a total of eight (8) trials (2 conditions × 4 images). The images were shown in a pseudo-random fixed order. Each small-form image was displayed for 5 s and was always preceded by an attention grabber shown in the center of the screen.

### 2.4. Data Reduction and Analysis

Eye-tracking data was processed in MATLAB (MathWorks, Friedrichsdorf, Germany) using the open-source data analysis tool Timestudio (http://timestudioproject.com, Nyström et al., 2015), version 3.12. The settings and source code can be downloaded with uwid: ts-53a-6ef within the TimeStudio environment.

Infants' gaze was analyzed in four areas of interest (AOI). Each centered over one of the small forms stimuli. AOIs subtended a visual angle of 13 × 19 (see Figure 1A). We measured infants' looking-time in each AOI. A minimum of 20% looking-time (1s) to the AOIs was required for a valid trial. A total of 67% of trials (107 out of 160) were included in the final analysis. The mean number of included trials per child in each of the four conditions was 2.7 (90°), 2.8 (60°), 2.4 (40°), and 2.8 (10°), respectively. For each valid trial, we calculated a proportion score as the proportion of looking-time in the target AOI to the total looking-time in all four AOIs. Thus, if infants' looking-times were equally distributed to all four AOIs we would expect a proportion score of 0.25.

We evaluated infants' looking time within and between conditions by means of Bayesian *t*-tests. Although Bayesian and standard *t*-tests often agree on which hypothesis is better supported by the data, the former has the advantage of allowing for a quantification of the evidence from the data in support of an experimental effect (Rouder et al., 2009; Wetzels et al., 2011). Our approach thus aligns with recent recommendations for psychological science to reduce the reliance on standard null-hypothesis testing and their accompanying *p*-values (Cumming, 2008). For each test we report a Bayes Factor (*BF*_10_) for the alternative hypothesis (*H*_1_) over the null hypothesis (*H*_0_) together with an effect size (δ)^1^ followed by a 95% confidence interval for the effect size in brackets. The Bayes Factor allows for the evaluation of the evidence for *H*_1_ compared to *H*_0_. If *BF*_10_ = 1 there is equal evidence for *H*_1_ and *H*_0_. A *BF*_10_>1 indicates more evidence for H1 than H0 while *BF*_10_ < 1 indicates the opposite^2^. All reported tests were performed in JASP (JASP Team, 2018) with the default Cauchy prior width of 0.707. We, however, verified that the same interpretation of the results held both with a wide (1) and an ultra-wide (1.5) width of the Cauchy prior^3^.

## 3. Results

### 3.1. Discrimination

**Figure 2:**
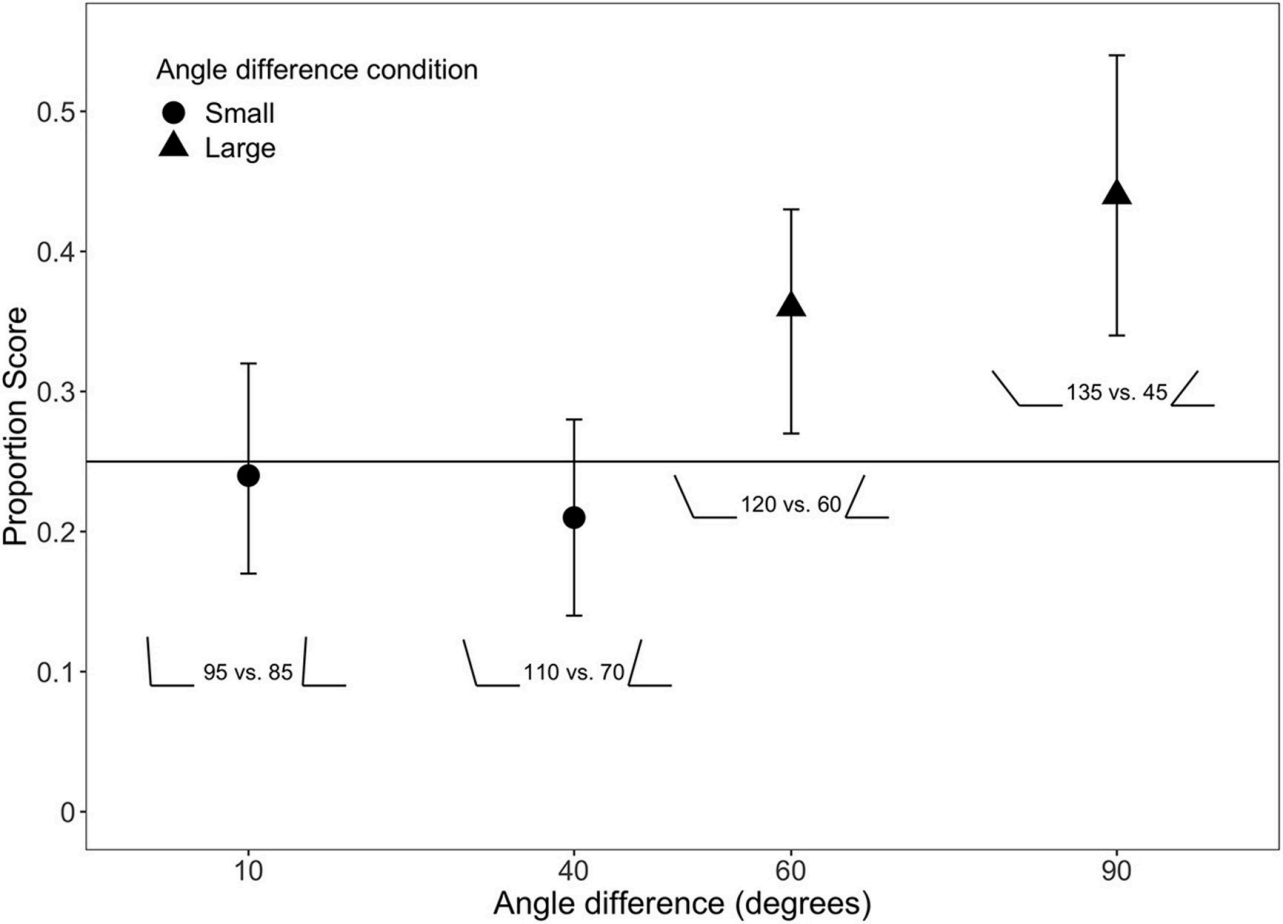
Mean proportion score in the four conditions. Angles show the two to-be-compared angles in each condition. Horizontal line denotes chance level. Vertical bars denote 95% BCa bootstrapped confidence intervals for the mean.

We evaluated infants' ability to discriminate between the four small forms by testing if the proportion score for their looking time differed from chance (*H*_0_ = 0.25). The results showed that participants in the *Large* condition could reliably identify the target angle above chance for both the 90° (*M* = 0.46, *SD* = 0.14; *BF*_10_ = 30.7, δ = 1.2[0.35, 2.18]) and 60° (*M* = 0.36, *SD* = 0.13; *BF*_10_ = 2.6, δ = 0.70[0.01, 1.45]) angle difference. Participants in the *Small* condition, however, failed to identify the target angle for both the 40° (*M* = 0.23, *SD* = 0.12; *BF*_10_ = 0.36, δ = *-*0.14[*-*0.73, 0.42]) and the 10° condition (*M* = 0.23, *SD* = 0.10; *BF*_10_ = 0.37, δ = *-*0.17[*-*0.74, 0.39]). In fact, in both of the Small conditions the Bayes' factor suggested evidence in favor of the null hypothesis. In the Large condition 90 and 89% of participants had proportion scores larger than 0.25 for the 90- and 60° angle difference, respectively. In the Small condition the corresponding proportions were 40 and 30% for the 40- and 10° angle differences, respectively. These results are summarized in Figure 2.

On average infants spent 3.0 s (*SD* = 0.91) looking at the four AOIs on each trial. There was no difference in looking times between the Large and Small conditions (*BF*_10_ = 1.2, δ = *-*0.57[*-*1.47, 0.18]).

### 3.2. Discrimination Over Time

To gain further insight into how the discrimination process unfolds over time, we analyzed which AOI infants looked toward first and how their preference for the deviant form evolved during the stimulus presentation. We analyzed which form infants directed their first gaze toward to investigate if infants have a very early preferences for the deviant form already at stimulus onset. This analysis showed that the proportion of first gazes toward the target form did not differ from 0.25 in any of the four conditions (BCa bootstrapped CIs overlapped 0.25 in all conditions). Thus, infants had no strong initial preference for the target form.

**Figure 3:**
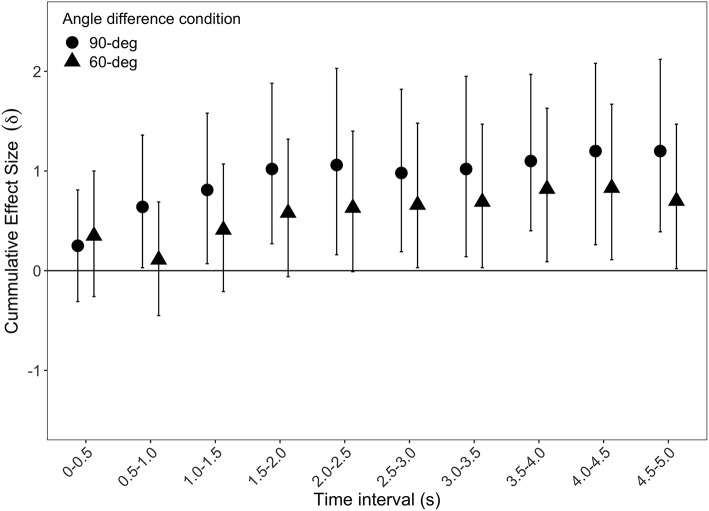
Median cumulative effect size as a function of time interval for the 60- and 90° conditions. Vertical bars denote 95% confidence intervals of the effect size.

To investigate the temporal dynamics of the preference formation for the target form, we divided each trial into ten segments of 500 ms each. Because infants in the 40- and 10° conditions never formed a preference for the deviant form, we only included the 90- and 60° conditions in the analysis. We investigated how long it took infants to form a stable preferences for the deviant form by running separate Bayesian *t*-tests for each of the ten intervals in each of the 60- and 90° conditions. In contrast to classical frequentist statistics, there is no commonly agreed upon cutoff for when the size of a Bayes factor should indicate that an effect is present. However, using the 95% confidence interval for the posterior distribution of the effect size it is possible to determine when it is highly likely than an effect is present (i.e., δ > 0). We therefore considered a preference to have been formed from the first interval for which the confidence interval of the effect size did not include zero. In the 90° condition this occurred in the 0.5–1.0 s interval (*BF*_10_ = 2.3, δ = 0.63[0.003, 1.35]) while infants in the 60° condition required an additional 2.5 s (3.0–3.5 s interval; *BF*_10_ = 2.5, δ = 0.69[0.01, 1.45]). Figure 3 illustrates these results.

## 4. General Discussion

In the present study, we aimed to bridge two lines of previous research that have evaluated infants' and children's ability, respectively, to discriminate between small forms based on geometric properties. Using eye-tracking, we investigated if 10-month-olds could discriminate between a set of small forms based on geometric properties in a deviant-detection paradigm and analyzed how the discrimination process unfolded over time. By manipulating the difficulty of the discrimination task, we also investigated the limit of this ability.

Our participants had a preference for the target form when the difference in angle size was 90 and 60° but not when the difference was 40 and 10°. These results suggest that infants, similar to young children and adults, can discriminate between small forms based on geometric properties in a deviant-detection paradigm, but only when the difference on a given property is sufficiently large. We thus extend prior findings showing that infants can extract an invariant geometric property over repeated presentations (Cohen and Younger, 1984; Slater et al., 1991) to show that they can also discriminate in a deviant-detection paradigm using the same type of information.

For the core cognitive system argument, it is important to show both that an ability to use geometric information is universal and that infants, children, and adults alike can solve similar geometric tasks. Previous studies have demonstrated that adults from cultures with no formal education in geometry - the Munduruku, living in the Amazon - and children as young as 4 years of age can make discriminations in the same paradigm, albeit somewhat less accurate than adults from western cultures (Dehaene et al., 2006; Izard et al., 2011). The demonstration in the present study that 10-month-old can make the same type of discrimination as adults and children is thus consistent with, and provides further circumstantial evidence for, the idea of a core system of geometry (Dehaene et al., 2006). To further extend what we know about the ontogenesis of a core system of geometry, it will be an important task to map out the developmental trajectory, from early infancy into adulthood, for this ability. By showing that the same type of paradigm, a deviant-detection task, can be used similarly at all ages, our study provides the means to conduct such an investigation. Note that although we have reasons to believe that infants in our study indeed respond to geometric information specifically, our effects are similar to results shown for other quantitative dimensions (e.g., numerosity and object size, Brannon et al., 2006; Libertus and Brannon, 2010). Some researchers have argued that such similarities over various types of magnitudes are indicative of a more general analog magnitude system (e.g., Walsh, 2003; Lourenco and Longo, 2010). Our study was not designed to discriminate between these two possibilities, but it is an essential question for future research to investigate if a core cognitive system for geometry is indeed a separate system or a feature of a general magnitude system.

It is reasonable to assume that an ability to discriminate between small forms has a limit on its acuity. In fact, previous research (Bonny and Lourenco, 2015) has shown that by 4 years of age, there is individual variability in geometric deviant-detection paradigms that correlate with the estimation of area. Thus, even in infants, detecting a difference between two small forms might require a minimum difference on a geometric property. It was not a priori evident if, or when, infants could no longer discriminate between target form and the deviant form because no other study has manipulated the difference in this manner. While the infants' in the present study could reliably detect differences in the 60° condition, they failed to do so in the 40° condition. In terms of a difference in angle, our results thus indicate that the limit of 10-month-old infants' ability to discriminate is to be found somewhere between 60 and 40° (or a corresponding difference in the distance between endpoints that lies between 4.53 cm [4.32 visual degrees] and 3.05 cm [2.9 visual degrees]). A caveat to this conclusion is that discrimination of small forms might be subject to hysteresis effects similarly to numerosity discrimination (cf., Odic et al., 2014). In that case, presenting an easier item first (e.g., 90°) might also allow infants to discriminate an even more difficult item (e.g., 40°) than the 60° items presented in the present experiment. The eye-tracking method developed here gives ample opportunity for future research to investigate this limit in greater detail at various points during development.

To our knowledge, no previous study has investigated how the process of discriminating small forms unfolds over time. Our data indicated that infants had no strong initial preference for the deviant form, as noted in the proportion of first gaze shifts toward the target form not differing from chance in any of the four conditions. Instead, it took infants 1–3.5 s depending on condition before a stable preference was formed. As illustrated in Figure 3, and by our analysis, a stable preference was, on average, formed well within 3.5 s in the 60° condition and within 1.0 s in the 90° condition. Note, however, that there was no difference between the two conditions at any time point (all *BF*_10_ ≤ 1). Accordingly, it is possible that the difference in the time it took for the preference to become stable in the 60- and 90° conditions, respectively, is due to more noisy data in the former than the latter condition. Future studies, will need to examine these differences in greater detail.

What might be the underlying psychological process that infants are engaged in during the discrimination process? We speculate that the process may take one of two general forms. One possibility is that the deviant form acts as a perceptual pop-out that drives attention (Adler and Orprecio, 2006). Another option is a process that accumulates evidence over time, similar to what is suggested by the drift-diffusion model (DDM, Ratcliff and Rouder, 1998). The DDM is originally a model of two-alternative forced-choice tasks that assumes a decision between two choices is made by accumulating evidence for one or the other of the alternatives over time until the evidence in favor of one of the options exceeds a threshold. In adults, this model has been able to predict the relationship between visual fixations and choice for multialternative choice situations (Krajbich and Rangel, 2011). Although the design of our study does not allow for any definite conclusions about which process infants are engaged in there is some evidence indicative of an accumulation process. More specifically, in contrast to what would be expected from a pop-out mechanism, infants' first gaze was not reliably directed toward the deviant form. Importantly, our eye-tracking paradigm makes it possible for future studies to tease these possibilities apart by, for example, investigating infants' gaze behavior as the number of distractors in the task increase.

We manipulated the difference between forms in terms of angle size. This manipulation, of course, simultaneously changes other properties of the forms (summarized in Table 1). Although this feature of the task makes it difficult to ascertain which dimension infants are discriminating on, two pieces of evidence indicate that they might indeed use differences in angle size. First, we can rule out that infants are using the enclosed area because this dimension is not discriminative between the target-form and the deviant form in any condition. Second, both previous research (e.g., Slater et al., 1991) and pilot data from our lab shows that when endpoint distance is controlled to be incongruent with angle size (by changing the length of the rays), infants still respond to angle size. We note, however, that controlling for one property (e.g., endpoint distance) often confounds another property (e.g., line length) with angle size (see also Cohen and Younger, 1984; Slater et al., 1991). It is, therefore, an important task for future research to further disentangle exactly what property infants use to discriminate.

## 5. Conclusions

Using eye-tracking, we investigated 10-month-old infants' ability to discriminate between small forms based on geometric properties in a deviant-detection paradigm. By investigating the possible presence of such abilities in infancy the present study extended previous research in three ways. First, we showed that infants could make similar discriminative judgments as children and adults, which is consistent with the idea of a core cognitive system for geometry. Second, by manipulating the differences between the to-be-discriminated forms, we were able to give a first crude estimate on the limit, in terms of acuity, on infants' ability to discriminate between small forms. Finally, we provided the first demonstration of how the discrimination process unfolds over time.

## Ethics Statement

This study was carried out in accordance with the recommendations of Regionala Etikprövningsnämnden with written informed consent from all subjects. All subjects gave written informed consent in accordance with the 1964 Declaration of Helsinki. The protocol was approved by the Regionala Etikprövningsnämnden.

## Author Contributions

ML, MR, BK, and GG planned the study. ML supervised the data collection and analyzed the data. ML and MR drafted the first version of the manuscript, to which all authors provided critical comments. All authors approved the submitted version of the manuscript.

### Conflict of Interest Statement

The authors declare that the research was conducted in the absence of any commercial or financial relationships that could be construed as a potential conflict of interest.

## Footnotes

^1^We report the standardized effect size δ, implemented in JASP and defined as δ = (μ−μ_0_)/σ and δ = (μ_2_−μ_1_)/σ for one-sample and two-sample tests, respectively (Rouder et al., 2009).

^2^One convention for how to interpret the support for the alternative hypothesis provided by *BF*_10_ is given by Wagenmakers et al. (2017). This convention suggests that a Bayes factor offers “extreme” (*BF*>100), “very strong” (30 < *BF* ≤ 100), “strong” (10 < *BF* ≤ 30), “moderate” (3 < *BF* ≤ 10), “anecdotal” (1 < *BF* ≤ 3), or “none” (*BF* = 1) evidence.

^3^We also verified that applying standard *t*-test with an alpha level of 0.05 did not change the interpretation of the reported results.
